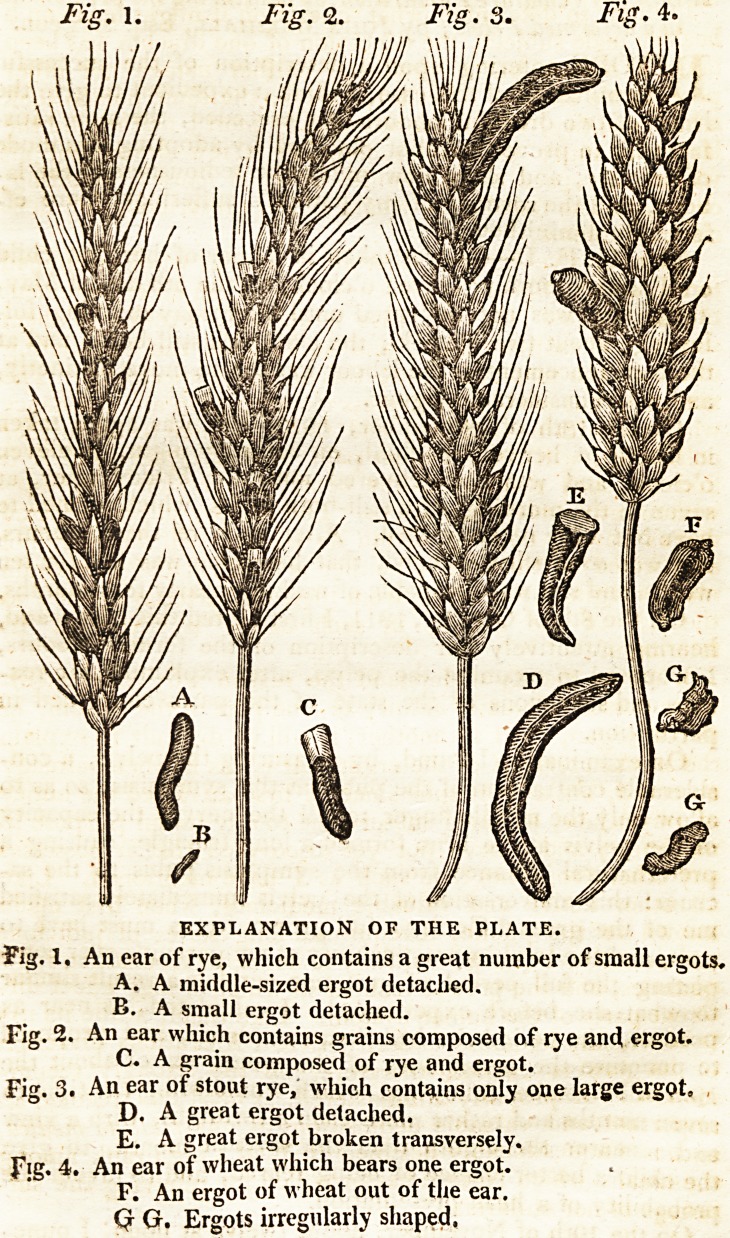# A Dissertation on the Natural History and Medical Effects of Secale Cornutum, or Ergot

**Published:** 1814-08

**Authors:** Oliver Prescot


*For the Medical and Physical Journal.*

### *A Dissertation on the Natural History and Medical Effects of Secale Cornutum, or Ergot;* by Oliver Prescot, A.M. Read at the annual meeting of the Massachusett's Medical Society, June 2, 1813.

AMONG the useful and important articles with which the *materia medica* of our country has lately been enriched, *one* has claimed an extraordinary degree of attention, from its being endued with singular and valuable properties, such as are denied to every other medicine with which we are acquainted—that of operating exclusively upon the *uterus.*

The recency of its introduction to medicinal use, will, I presume, render what little information I can give respecting it acceptable. Permit me, therefore, to attempt a brief detail of what has been discovered relative to its origin, its generic form and character; and at the same time consider, as far as the occasion will allow, the deleterious effects that have been ascribed to it, and the medicinal purposes it is calculated to subserve.

This production is generated by a peculiar disease, which occasionally affects the grains of rye, and is one of the four diseases of plants enumerated by Linnæus, and by him denominated *clavus;* some naturalists call it *clavus secalinus*, or *mater secalis*, others *secale cornutum*, and *secale luxurians.* The French term this production *bled cornu*, *seigle ergote*, or *orgot.* This disease very often attacks the rye in France. In the province of Salonia, more especially, it is very predominant; and in such seasons as are very moist, is occasionally seen in Great Britain and other parts of Europe. The rye in this country also, is so liable to the same disease, that, in our new settlements there is always, I believe, more or less of it to be found in this grain; but is more rarely to be discovered on fields that have been kept in a state of constant cultivation, for a considerable number of years; as those in the vicinity of Boston, and other towns on the seaboard.

The earliest account of this diseased rye is probably that of M. Dodart, in 1676; the latest I have seen is a memoir of l'Abbé Tessier, read before the Royal Medical Society at Paris, in 1776. To this last I am principally indebted for the following facts relative to its natural history, most of which accord with my own observations.

This diseased grain, which I shall call *ergot*, is found in the ear of the rye, in greater or less quantity, according to the season, and its situation. Its form is ordinarily crooked and long; it projects much from the *glume;* is larger in the middle than at the extremities, which are sometimes blunt, and sometimes pointed. It is seldom round in its whole length, there being generally three angles, and certain longitudinal lines, extending from one end to the other. In many grains, particularly the largest, there are small cavities, supposed by some to be occasioned by insects, by others by the sun. Its external colour is violet of different degrees of intensity, which encloses a dull white substance of a firm consistence, from which the external coat does not separate itself even after long boiling.

A grain of ergot breaks short, like a dry almond, and has nothing disagreeable either in its odour or taste; the grains are of different size, and vary in their length. Some are less than the grains of rye themselves, while others are eighteen or nineteen lines in length, and two or three in thickness; but the length is more usually ten or twelve lines. Sometimes they are short, and at the same time large; but these are not of an ordinary form.

When the ergot is large, there are generally but few upon an ear, and the grains of rye, on the same ear, are fine and healthy, and the whole plant vigorous; on the contrary, when the grains of ergot are small, there are many on an ear, and the stalk is less strong and thrifty. There are commonly found four or five of these grains upon one ear, frequently ten or twelve, and sometimes even twenty. The grains of rye in those ears which have many ergots are never good, but are shrunk, and covered at their superior extremity, with a black powder.

This production, if exposed to the air, dries readily, and becomes less in size, and very light. A measure of it, that holds fourteen pounds of rye, will weigh but nine pounds.

It is occasionally found on *wheat*, but on the ears of this grain it is always short, though thick and well nourished; the quantity, however, produced by this plant is very inconsiderable.

On many ears of rye, there are to be found grains composed of rye and ergot, the portion ergotted makes sometimes one-half, and sometimes only one-third of the grain, and is that part within the husk, while that part which is rye is most distant from the ear. These grains, if planted, will not vegetate, the germ being destroyed. Winter and spring rye are, as far as has been observed, equally liable to this disease.

Much time and attention have been devoted by different naturalists, to ascertain the cause of this production in rye. Some, from the circumstance that there is more produced in rainy seasons, and in wet grounds, have attributed its formation to the moisture of the air and the earth; others believe it to proceed from the grains having been pierced by insects; while others have regarded it as a *mole*, occasioned by a faulty fecundation. This last opinion is more probably correct, for nothing has been found to contribute so much to its production, if the soil be moist, as a storm of rain falling upon the grain when in bloom.

There will always be more of it found on the borders of fields, than in other parts, where the soil is less beaten and more mellow. The humidity being equal, those fields are most infested with it, which have been newly turned up.

The soil and climate of Sologne are so peculiarly adapted to the growth of this substance, that it is said to produce more of it than all France beside; for, in some years, not less less than one-fourth of all the grain raised in that province, is ergotted. In this district and its vicinity, there has, at different periods, prevailed among the peasants, a very malignant and mortal disease, which is characterized by a dry gangrene in some one of the extremities, sometimes in all of them, which has been generally ascribed to their living upon bread made of the ergotted rye.* This bread, M. Dodart informs us, does not differ, in regard to taste, from ordinary bread; is more particularly pernicious when new; but its effects are not observed until it has been eaten a considerable time. According to the observations of M. Noel, the ergot loses its deleterious qualities altogether, after having been kept a few months in sheaf: and writers all agree in this, that the disease it is supposed to induce is prevalent only at the conclusion of harvest, and ceases entirely before the commencement of winter.

Besides this spontaneous gangrene of the limbs, Hoffman and other writers have attributed also to its use another species of disease, which prevailed at different periods, in various parts of Europe, attended by convulsions and spasmodic affections. But these are now generally considered as originating from other causes.

In France, many experiments have been made on animals, to prove its malignant effects, and numerous communications have been published, shewing its noxious properties; but I believe it has never been considered, by any of these writers, as capable of subserving any medicinal or other useful purpose.

Some few empirics, however, it is said, have long known that the ergot would expedite lingering labour. But these ignorant pretenders bestow upon their nostrums such extravagant encomiums, and their impositions upon the credulity of the public are so numerous and frequent, that no credit whatever can be attached to their recommendations. Most of their mighty secrets, when disclosed, prove altogether inert; or at best very incompetent to effect the purposes for which they are intended. Their powder, to promote delivery, was consequently derided, and was thought by the faculty to be unworthy of serious attention or regard.

The first information the public received, from a source entitled to credence, that this production was, in reality, endued with such an unexampled property, was through the

* For a particular account of this disease, and the method adopted for its cure, vid. Duncan's Med. Com. vol. ix. p. 78.

medium medium of the New-York Medical Repository,* by a letter from Dr. J. Stearns to Dr. Akerly. In this communication Dr. Stearns designates it by the appellation of *pulvis parturiens.*

Very soon after this publication, I procured a sufficient quantity for experiment, and have since frequently used it. With very few exceptions, its uniform effect is to stimulate the uterus to increased action, when administered in parturition. But I cannot say with Dr. Stearns, "I have never been disappointed in my expectations of its effects;" for I met this disappointment in the very first case in which I prescribed it. In that case, a neighbouring physician was attending the patient, the travail had progressed slowly, but in a regular manner, until the head of the fœtus was detruded so low in the pelvis, that the ear was perceptible to the touch, when the pains subsided, and had entirely ceased, some hours before I was summoned. One drachm was administered, in the form of decoction, at three separate doses, but without producing any effect, when the delivery was accomplished by the aid of the *forceps.*

Two similar cases have since occurred, in which the pains had totally ceased, toward the termination of labour, and in which parturient efforts could not be revived, by any quantity I thought prudent to administer. In one of these last, the patient took the decoction of more than two drachms in divided doses.

In four other patients, I had reason to doubt whether the pains were increased by its use, either in frequency or strength; but one dose only was given to either of them, for the irritable state of the stomach prevented its being repeated.

In every other instance, without exception, the effects of this prescription have been such as fully to demonstrate its powers "*ad partum accelerandum.*" The pains produced by it, when a full dose is given, are very peculiarly forcing, and the contractile effort of the uterus continues to that degree, that the fœtus is not suffered to retreat, but remains firmly retained where the last exacerbation of pain left it, until it recurs again. This incessant action will continue, if the delivery is not effected, for an hour or more, and when it subsides, the medicine, again given, will reproduce the same effects.

The frequency and violence of the uterine efforts, induced by the ergot, are not more extraordinary, than is its almost instantaneous operation. In twenty cases, I carefully noticed

* Vol. ii. p. 308.

the the precise time it required, to produce its customary effects. In two of them, the increased strength of the pains, and the continued action commenced in seven minutes from the time the decoction was taken; in one case it was eight minutes, in seven it was ten, in three eleven, and in three others it was fifteen minutes. In the four remaining cases, there was no apparent operation until twenty minutes had expired. In other cases, the time was not particularly noticed, but, as the twenty I have given were nearly in succession, it is probable they will shew the proportion as accurately as if the time in all had been precisely ascertained.

From this account of the manner in which the ergot usually operates, it will be readily conceived, by those who have not witnessed its effects, that it is a powerful agent, which requires prudent direction; but, when properly applied, will be highly useful, many times, to shorten a process, which, unaided, would prove extremely tedious and troublesome.

Before I had acquired sufficient experience of its effects, I imprudently used it once or twice when the pains were tardy and feeble, even in first labour, before the orifice of the uterus was much relaxed or dilated; it having been recommended to "produce all the beneficial effects of bleeding without inducing the debility." But it does *not* usually prove relaxing to the rigid fibre; its operation, therefore, subjected the patients to much unnecessary suffering. In one instance, no perceptible progress was made, by the continuance of forcible uterine efforts, during the space of an hour.

It is therefore important, even if the pains are feeble and unfrequent, to delay giving this stimulating drug, until considerable dilatation has taken place; to leave the business in its early stages to the slow and regular process of nature; and by the respite thus gained by the intervals from pain, preserve the strength and resolution of the patient for later and more painful efforts.

But if the labour should be long protracted, from the irregular action of the uterus, or the rigidity of the muscular fibres, these obstacles should be first removed by venesection; after which the ergot may be usefully employed, and its operation will be found mild and efficacious. But whenever recourse is had to venesection, the depletion should be copious, and the blood suddenly drawn from a large orifice, for no possible advantage will be gained by this operation, upon a plethoric subject, if the quantity taken be less than twenty ounces; and I have repeatedly taken thirty, before the necessary end could be accomplished.

I have never administered ergot in substance, but always in in the form of decoction, in the proportion of half a drachm to four ounces of water, of which one-third is taken at a time; if the pains are not sufficiently augmented in twenty minutes, then half the remainder is given; but a second dose is rarely required.

It will probably be found more beneficial in many cases to diminish the quantity to one large table spoonful, which, taken every ten minutes, will have the effect to increase the vigour of the pains, without producing such excessive and constant action, as is usual when the full dose is administered. I have lately directed it in this manner, and have been so much gratified with its more temperate, though efficient, action, that I shall hereafter prefer the smaller to the larger quantity.

It has been suggested, by a writer in the New-England Journal of Medicine and Surgery, that the death of the infant is a more frequent occurrence, in cases in which the ergot has been employed, than where its agency has not been used. If this is indeed the case, it forms at once an insuperable objection to its use, except in cases where its safety is well defined; and the subject certainly demands deliberate attention and serious inquiry. For myself, it is, I conceive, rather questionable, whether more injury would result to the child "from unceasing pressure for several minutes, and occasionally for half an hour or more," than for a much more tedious process, in which the pressure is reiterated, and the head permitted to retreat after each successive effort. But, in a matter of such importance, we ought not to be governed by conjecture; but should adopt or reject it, as its beneficial or destructive operation is tested by experiment. My own experience has been such, as to persuade me, that the above suggestion is unfounded. It is true, that in twenty-two cases of first labour, in which this medicine had any effect, I lost four children; and in thirty-five where it was given to women, who had been previously delivered, I have lost one. But all these deaths were attended with such circumstances, as fully to exculpate the ergot from any agency in the event. And, when it is recollected that this medicine is not used, except in cases that are long protracted, or are likely to prove tedious and troublesome, it will not be thought, I conclude, that this unfortunate event happened more frequently, or in greater proportion to the whole number of cases, than might reasonably have been expected, had this medicine not been prescribed. But exclusive of any injurious effects, which may result to the infant, the ergot requires much more caution with respect to its use, in cases of first labour, than in others; for, owing to the usual tension and rigidity of the parts, the protruding progress will not be accelerated, in any reasonable proportion to the additional pain and suffering it produces. It is also too active and powerful an agent, to be safely directed by an ignorant or unexperienced accoucheur; and, before dismissing the subject, I most cordially join in cautioning those who have not been in the practice of using it, and witnessing its operations, to be wary how they employ it until the muscular fibre is properly relaxed, and the *os uteri* considerably dilated. This caution is also more especially necessary, if they are not positively certain that the presentation is natural, as well as "that there are no preternatural obstructions, to prevent delivery; as the violent pain, and almost incessant action, which it frequently induces, in the uterus, precludes the possibility of turning" the fœtus.

Dr. Beekman is said to have succeeded in a case of amenorrhœa, by giving one drachm of the ergot in decoction. In consequence of this recommendation, I tried its effects in one case of partial obstruction, by giving it, first in a dose of one drachm; at the next period the same patient took two drachms, but without the desired effect. And from analogy, I should conclude, that it was unadapted to this complaint. The tendency of its operation is, I conceive, to constringe the uterine fibres, and lessen the caliber of its blood-vessels; for, when given to parturient patients, there has been no instance, within my knowledge, of undue hemorrhage after delivery, although several, who have taken it, had been previously accustomed to profuse discharges. The lochia also, have occasionally been so much diminished, after its use, as to excite apprehension for the event. In two cases this discharge entirely ceased on the second or third day after delivery, and did not re-appear during the month; but no puerperal complaint was induced, nor was their recovery delayed by this incident.

The uniform operation of the ergot to restrain uterine hemorrhage, has been noticed by other physicians. It has in consequence frequently been prescribed, a little previous to the birth of the child, or immediately after, to patients that have been accustomed to flow immoderately at such times, and it has always proved an effectual preventive.

This singular property of the ergot, to diminish the enlarged cavity of the uterus, is never more strikingly exemplified, than when its agency is employed to restrain those floodings which sometimes appear in the early months of pregnancy, when the action of gestation has ceased, and abortion must follow. In such cases it speedily excites in the uterus such energetic action, that its contents are soon expelled, and the hemorrhage ceases.

In order to determine what operation it might have on a healthy male subject, the decoction of one drachm has been taken at a dose, but it produced neither nausea nor other perceptible effect. After a few days, the same person took a like quantity, which proved equally inert; neither did the larger quantity of two drachms, at a few doses, but all within the space of two hours, occasion nausea, vomiting, or pain in the female, to whom it was prescribed, for deficient catamenia.

Its operative powers, therefore, appear wholly confined to the uterine fibres, when lengthened from an enlargement of that viscus. In such case it speedily excites in them strong contractile action, and so long as the stimulating effect of the medicine lasts, this action is unceasing. The uterus is thus made to compress closely, upon any substance whatever within its cavity, and this resistance to its further collapsing, will cause violent pain in that organ; but if it find no such resistance, the contractile action progresses without any uneasy sensations. The healthy unimpregnated uterus having nothing within its cavity, will therefore not be affected by the ergot; neither is it calculated to restrain menorrhagia, proceeding from increased arterial action; as the size of the uterus, in such cases, is nearly at its minimum.

Until we clearly understand the reason why some medicines possess a greater affinity to one part of the system, or to one organ, than to another, it will be difficult to explain the *modus operandi* of the ergot. It is, as has been already observed, but a short time since it first attracted the notice of physicians, as being subservient to any useful purpose in medicine; and I have not yet discovered that it possesses any other properties than such as I have mentioned. Like all other active and valuable medicines, when first made known to the public, it requires a long series of judicious and attentive experiments, fully to develope its character, its qualities, and the precise manner in which it may affect different parts of the human system. Like them, while its use is beneficial, its abuse is destructive. A cautious direction of its powers cannot, therefore, be too strongly recommended. If properly administered, it must be esteemed an important and valuable acquisition to our *materia medica*, and is unquestionably destined to hold a high rank among the means which Nature has provided for relieving the sufferings of her children.

Plate copied from the one annexed to l'Abbé Tessier's Memoir in Histoire de la Société Royale de Médecine. Tom. 1. pars 2d. p. 418.

## Figures and Tables

**Fig. 1. Fig. 2. Fig. 3. Fig. 4. f1:**